# Heart failure with preserved ejection fraction: an update on pathophysiology, diagnosis, treatment, and prognosis

**DOI:** 10.1590/1414-431X20209646

**Published:** 2020-06-05

**Authors:** Chao Ma, Huan Luo, Lei Fan, Xiaoyan Liu, Chengshan Gao

**Affiliations:** 1 Berlin Institute of Health Center for Regenerative Therapies & Berlin - Brandenburg Center for Regenerative Therapies (BCRT), Charité - Universitätsmedizin Berlin, Campus Virchow Klinikum (CVK), Berlin, Germany; 2 German Center for Cardiovascular Research (DZHK), Partner Site Berlin, Berlin, Germany; 3 Klinik für Augenheilkunde, Charité-Universitätsmedizin Berlin, Corporate Member of Freie Universität Berlin, Humboldt-Universität zu Berlin, and Berlin Institute of Health, Berlin, Germany; 4 Department of Orthopedic Surgery, Henan Provincial People's Hospital, Zhengzhou, Henan, China; 5 Department of Cardiovascular Surgery, Second Affiliated Hospital of Zhengzhou University, Zhengzhou, Henan, China

**Keywords:** Heart failure with preserved ejection fraction, Heart failure with reduced ejection fraction, Heart failure, HFpEF, HFrEF, Pathophysiology

## Abstract

Heart failure (HF) with preserved ejection fraction (HFpEF) is a clinical syndrome in which patients have symptoms and signs of HF with normal or near-normal left ventricular ejection fraction (LVEF ≥50%). Roughly half of all patients with HF worldwide have an LVEF ≥50% and nearly half have an LVEF <50%. Thanks to the increased scientific attention about the condition and improved characterization and diagnostic tools, the incidence of HF with reduced ejection fraction (HFrEF) dropped while that of HFpEF has increased by 45%. HFpEF has no single guideline for diagnosis or treatment, the patient population is heterogeneously and inconsistently described, and longitudinal studies are lacking. To better understand and overcome the disease, in this review, we updated the latest knowledge of HFpEF pathophysiology, introduced the existing promising diagnostic methods and treatments, and summarized its prognosis by reviewing the most recent cohort studies.

## Introduction

Heart failure (HF) is when the heart is unable to pump sufficient blood to maintain blood flow to meet the body's needs. HF is a common, costly, and potentially fatal condition (1). In 2015, it affected about 40 million people globally (1). Overall, around 2% of adults have HF, and in those over the age of 65, this increases to 6–10% (1).

In the past, heart failure with reduced left ventricular ejection fraction (HFrEF) was the most commonly diagnosed clinical entity in HF patients. However, with the improvement of diagnostic tools, especially with the introduction of new echocardiography modalities, HF has recently been classified into three subtypes, namely HFrEF, heart failure with preserved ejection fraction (HFpEF), and HF mid-range ejection fraction (HFmrEF), according to the ejection fraction, natriuretic peptide levels, and the presence of structural heart disease and diastolic dysfunction (2).

HFpEF is a vital component of HF. Patients with HFpEF have significant morbidity and mortality but, unlike HFrEF,there are currently no effective validated therapies. In addition, HFpEF is poorly investigated (3). To better understand the mechanisms underlying this disease and help scientists to explore future therapies, we updated here the latest knowledge of the pathophysiology, diagnosis, treatment, and prognosis of HFpEF (3).

## Pathophysiology

### LV structure and remodeling

The structural remodeling that often occurs in HFpEF differs dramatically from that in HFrEF. Early descriptive studies in HFpEF suggested that concentric left ventricular (LV) hypertrophy with normal chamber size was typical (4). However, several patients with hemodynamic evidence of HF do not have structural remodeling of the heart but have normal LV geometry (4). Thus, the absence of structural heart disease does not exclude the diagnosis of HFpEF. Many, but not all, patients with HFpEF exhibit a concentric pattern of LV remodeling and a hypertrophic process that is characterized by the following features (4): 1) normal or near-normal end-diastolic volume; 2) increased wall thickness and/or LV mass; 3) increased ratio of myocardial mass to cavity volume; and 4) increased relative wall thickness (RWT). The RWT is defined as either 2 X (posterior wall thickness) / (LV diastolic diameter) or as (septal wall thickness + posterior wall thickness) / (LV diastolic diameter). At the structural level, cardiomyocytes in HFpEF are thicker and less elongated than in HFrEF, and collagen content is increased compared with control populations (4). By comparison, patients with HFrEF typically exhibit a pattern of eccentric remodeling with an increase in end-diastolic volume, an increase in LV mass but little increase in wall thickness, and a substantial decrease in the ratios of mass to volume and thickness to radius (4).

### LV diastolic limitations

Diastolic dysfunction is defined as the inability to fill the ventricle to an adequate preload volume (end-diastolic volume; EDV) at acceptably low pressures (5). Diastolic function is often conceptualized as the totality of an active process of pressure decay (relaxation) during early diastole related to myofilament dissociation and calcium reuptake, and ‘passive’ stiffness associated with the viscoelastic properties that are governed by mechanical changes from the sarcomere to extracellular matrix, chamber, and pericardium (5). Diastolic dysfunction and HFpEF are not synonymous terms (5). Diastolic dysfunction indicates a functional abnormality of diastolic relaxation, filling, or distensibility of the LV, regardless of whether the LVEF is normal or abnormal and whether the patient is symptomatic or not (5). Thus, diastolic dysfunction refers to unusual mechanical properties of the ventricle (5). HFpEF denotes the signs and symptoms of clinical HF in a patient with a normal LVEF and LV diastolic dysfunction (5). Diastolic dysfunction alone is essentially part of normal human aging and is seen in many people that do not or never will have HFpEF. However, the presence of diastolic dysfunction is a risk factor for developing HFpEF (5).

Most, although not all, studies have demonstrated that the rate of LV pressure decay during isovolumic relaxation (time constant τ) is prolonged in HFpEF (6). The minimal LV diastolic pressure or completion of relaxation normally occurs by 3.5 times the value of τ (normal τ <45 ms) after the mitral opening (6). However, when the heart rate increases, the left ventricle must enhance relaxation to allow for faster pressure decay. In HFpEF, this enhancement is lost, contributing to LV and left atrial (LA) pressure elevation (6).

Delayed relaxation is, however, only part of the problem in early diastole in HFpEF. The healthy left ventricle functions as a ‘vacuum cleaner’ that prevents LA hypertension by enhancing suction in response to increases in venous return (7). Studies have shown that the ‘vacuum cleaner’ function of the LV in patients with HFpEF is lost, especially when the heart rate is elevated. The filling of the LV can only rely on the high pressure of the LA (7).

Ventricular passive diastolic stiffness is also an essential determinant of the increase in LV filling pressures in HFpEF (8). Most, but not all, studies have shown that, on average, LV end-diastolic stiffness is increased in patients with HFpEF (8). In the absence of endocardial or pericardial disease, diastolic LV dysfunction results from increased myocardial stiffness. Two compartments within the myocardium regulate its diastolic stiffness: the extracellular matrix and the cardiomyocytes. A stiffness change within one compartment is also transmitted to the other compartment via matricellular proteins (8). Previous studies in the last 15 years have pointed to the importance of determinants within the cardiac myocytes, particularly the sarcomeric macromolecule titin in diastolic ventricular passive stiffness (8). The (giant) elastic sarcomeric protein titin is the dominant regulator of myocardial passive tension, and thus of the cardiomyocyte-derived stiffness (8). Up to 80% of left ventricular passive stiffness may be explained by titin, especially when sarcomere lengths are still within physiological boundaries, while in over-stretched sarcomeres the contribution of the extracellular matrix becomes more dominant (8). Titin regulates cardiomyocyte stiffness at the transcriptional and post-translational levels. At the transcriptional level, titin shifts from its compliant isoform N2BA toward its stiff isoform N2B have been postulated to contribute to diastolic dysfunction in HFpEF (8). Post-translational modification of the titin N2B segment by protein kinase A (PKA)- and G (PKG)-mediated phosphorylation has been shown to change cardiomyocyte passive tension (8). Diastolic intracellular calcium handling is a major determinant of LV relaxation. Dephosphorylated phospholamban (PLN) is an inhibitor of sarcoplasmic/endoplasmic reticulum Ca(2+)ATPase 2a (SERCA2a), but PKA-catalyzed (or CaMKII) phosphorylation of PLN results in the dissociation of PLN from SERCA2a, thus activating this Ca^2+^ pump and augmenting SERCA2a activity (9,10). Cardiomyocyte Ca^2+^ accumulation in the absence of concomitant enhancement of SERCA activity leads to elevated diastolic Ca^2+^, Ca^2+^ transients with preserved or enhanced amplitude, and slower Ca^2+^ reuptake kinetics with impaired relaxation. The inability of SERCA to expeditiously resequester Ca^2+^ becomes particularly evident at elevated stimulation frequencies, which may in part explain the chronotropic intolerance of the myocardium and reduced exercise capacity of HFpEF patients (11). Preclinical studies and clinical trials indicate that combining SERCA2a activation and Na^+^/K^+^‐ATPase (NKA) inhibition may increase contractility and facilitate active relaxation, improving systolic as well as diastolic heart function, both of which would be beneficial effects in the treatment of chronic HF (12). Researchers have proposed that the diverse comorbidities of HFpEF contribute to a systemic inflammatory state, which induces microvascular endothelial inflammation resulting in endothelial dysfunction, reactive oxygen species production, and reduced nitric oxide (NO) bioavailability (13). This finding provides a novel therapeutic target to improve diastolic stiffness and it is speculated to mechanistically tie together the loss of NO bioavailability in HFpEF with conventional risk factors including obesity, aging, and metabolic syndrome (13).

Diastolic dysfunction is not common in HFpEF. Diastolic dysfunction cannot be observed by echocardiography at rest in one-third of patients with HFpEF (14). Many patients with HFpEF in the early stages did not present an increase in LV filling pressure at rest (14). These patients usually have normal plasma levels of type B natriuretic peptide (BNP), which leads clinicians to make a false diagnosis of no HF (14). This can be explained since natriuretic peptides are released and produced in response to increased myocardial wall tension. HFpEF is characterized by hypertrophic hearts with a small LV cavity, and this structural abnormality in itself does not elevate end-diastolic wall stress much, as can be perfectly concluded from Laplace's law (15). In addition, obesity is associated with lower than normal BNP levels, and these findings may explain the reduction in BNP levels observed in patients with HFpEF (14).

Studies have shown that diastolic dysfunction in HFpEF does not appear to impair net LV filling, but this level of filling is at the expense of abnormal pressure elevation (4). In a prospective trial, aggressive treatment to reduce LV filling pressure in HFpEF was associated with a reduction in HF hospitalization (4,16). This further demonstrates that the importance of diastolic dysfunction in HFpEF should not be underestimated. Increased LA pressure can lead to dyspnea, secondary pulmonary hypertension, and atrial remodeling, which may make patients prone to right ventricular (RV) dysfunction and atrial fibrillation (14).

The most conspicuous and commonly present abnormalities in patients with HFpEF are related to diastolic dysfunction. This may present with impairments in relaxation, increases in chamber stiffness, or both. These abnormalities lead to an increase in LV filling pressures at rest or during exercise that causes dyspnea.

### LV systolic limitations

Although ejection fraction is the measure that is used most often clinically to assess systolic function, it is more appropriately viewed as a reflection of ventricular-arterial coupling (17). By definition, the LV ejection fraction (EF) and most indices of contractile function are normal or nearly normal in patients with HFpEF. However, EF is a poor and nonspecific index of contractile function. EF can be low owing to very high afterload despite normal contractility, or it can be normal even when contractile function is impaired when afterload is low. Multiple studies have shown that, despite relative preservation in LV EF, patients with HFpEF display subtle abnormalities in systolic function. Studies evaluating load-independent measures of chamber and myocardial contractile function have shown that there are decreases in systolic function in patients with HFpEF compared with age-matched healthy controls as well as asymptomatic hypertensives (17). This finding of impaired systolic function has been confirmed in numerous studies utilizing tissue Doppler and strain imaging techniques (17). These abnormalities are most conspicuously noted in longitudinal contraction and motion of the basal LV in the region of the mitral annulus (17).

Abnormalities in LV systolic properties are strongly associated with adverse outcomes in patients with HFpEF (18). Inability to augment systolic function also causes and worsens diastolic reserve in HFpEF (18). These relatively mild abnormalities in systolic function at rest become much more significant limitations during exercise, which further burden an already impaired heart. Prior studies have shown that the inability to augment cardiac output during exercise is related mainly to poor systolic reserve, where a contractile function cannot be supplemented during stress in a usual fashion. This limits the ability to augment forward stroke volume and reduces cardiac output and end-organ perfusion (18).

### Ventricular dyssynchrony

Ventricular dyssynchrony is a difference in the timing, or lack of synchrony, of contractions in different ventricles in the heart. Large differences in timing of contractions can reduce cardiac efficiency and is correlated with HF (19). Mechanical dyssynchrony is a term used to describe systolic and diastolic mechanical variability. A previous study has suggested that approximately 30% of patients with a narrow QRS have mechanical dyssynchrony. HFrEF patients exhibited increased systolic dyssynchrony compared to HFpEF patients despite a narrow QRS complex in addition to the more reduced diastolic and systolic function (19). Although electrical dyssynchrony (bundle branch block) is uncommon in patients with HFpEF, studies have shown that systolic and diastolic mechanical dyssynchrony is fairly prevalent (19). The magnitude of dyssynchrony is related to the extent of diastolic dysfunction and the magnitude of aerobic limitation (19). While therapies for dyssynchrony, such as biventricular pacing, provide benefits to HFrEF patients, no benefit is appreciable in HFpEF patients at this time (19). Evidence demonstrated that targeting the improvement of diastolic and systolic function instead of managing systolic dyssynchrony might be of great importance in the treatment of HFpEF (19).

### Atrial dysfunction and atrial fibrillation

The left atrium functions as an essential barrier between the LV and the pulmonary circulation, by facilitating LV filling through its conduit and booster functions and by shielding the pulmonary vasculature from full LV pressure oscillations in concert with the mitral valve apparatus (20). In a healthy heart, about 80% of LV filling occurs in early diastole, and the remaining 20% depends primarily on LA contraction. Studies have shown that early-stage HFpEF patients may be more dependent on LA contraction to achieve LV filling than healthy people (20). In the more advanced stages of HFpEF, progressive atrial dilatation and loss of atrial contractile reserve are more likely to occur, especially under stress (20). LA dysfunction in HFpEF is associated with a higher risk of HF hospitalization independent of potential clinical confounders, but not independent of LV strain and filling pressure. Impairment in LV systolic and diastolic function largely explain the association between impaired LA function and a higher risk of HF hospitalization in HFpEF (20).

Atrial fibrillation is common in HFpEF, identified at some point in two-thirds of patients, and its presence is associated with decreased exercise capacity, development and worsening of RV dysfunction, and increased mortality (21). The importance of LA function in HFpEF is underscored by observations that atrial fibrillation is very poorly tolerated in patients with HFpEF (21). Atrial dilatation precedes atrial fibrillation and is associated with chronic LV diastolic dysfunction as well as comorbidities commonly associated with HFpEF, such as obesity and disordered breathing during sleep (21). At this time, prospective data comparing rate and rhythm control strategies in HFpEF are lacking. Data indicate that impairments in LA function are also associated with adverse prognosis and a more significant burden of pulmonary hypertension in patients with HFpEF, even among patients in sinus rhythm without atrial fibrillation (21).

### RV dysfunction and pulmonary vascular disease

Roughly 70 to 80% of patients with HFpEF display pulmonary hypertension (22). As left atrial and pulmonary venous pressures increase due to diastolic dysfunction, this increases the pulmonary artery pressure through passive back-transmission of this hydrostatic pressure. With more advanced stages of HFpEF, there may also be changes in pulmonary vascular structure and function leading to a "precapillary" component where pulmonary vascular resistance increases (22). In patients with HFpEF, each 10-mmHg increment in pulmonary artery pressure is associated with a 28% increase in 3-year mortality (22). The chronic obstructive pulmonary disease commonly coexists with HFpEF, can worsen pulmonary hypertension, and also makes determining whether symptoms of dyspnea are primarily related to the heart or lungs (22). The presence of pulmonary hypertension (PH) in HFpEF is associated with adverse outcomes, including increased mortality and HF hospitalization rates (22). Reducing pulmonary artery pressure through diuretic use (which reduces LV and LA pressures) decreases HF hospitalizations in HFpEF (22), but other trials testing PH-specific therapies in HFpEF have failed to show a convincing benefit (22,23).

Prior studies have reported that RV dysfunction is present in HFpEF based upon non-invasive measures of RV shortening or systolic velocities (24). Studies have also demonstrated that RV dysfunction is common in HFpEF, seen in 20 to 35% of patients (24). Similar to what is seen in the left side of the heart, there is also RV diastolic and systolic dysfunction in HFpEF, at least in the more advanced stages of the disease. RV dysfunction seems to develop more in patients with lower LVEF, with more severe PH, and in patients with atrial fibrillation. The presence of RV dysfunction is a potent marker of increased morbidity and mortality, independent of the severity of PH in HFpEF (24). Deterioration in RV function was much greater than that seen in the LV over time. The development of RV dysfunction in HFpEF was associated with both prevalent and incident atrial fibrillation (AF), higher body weight, presence of coronary disease, higher pulmonary artery and LV filling pressures, and RV dilation. One study showed patients with HFpEF developing incident RV dilation had a nearly two-fold increased risk of death (adjusted hazard ratio 1.89, 95%CI: 1.01-3.44) (24). Therefore, among patients with normal LVEF and significant RV dysfunction, an advanced stage of HFpEF should be suspected (24).

### Pericardial restraint

The normal pericardium restrains ventricular filling, contributing to the elevation in intracardiac pressures that develop during conditions of increased venous return such as exercise (6). Patients with HFpEF characteristically develop marked increases in filling pressures with exercise or volume loading owing to diastolic dysfunction (6). Further study is required to determine whether pericardial restraint or enhanced diastolic ventricular interaction contributes to the pathophysiology of HFpEF, which would then raise the question as to whether surgical approaches to remove pericardial restraint might improve symptoms related to venous congestion (25). One recent study demonstrates that pericardial resection through a minimally-invasive percutaneous approach mitigates the elevation in LV filling pressures with volume loading in both normal animals and a pig model with diastolic dysfunction (25). Further study is warranted to determine whether this method is safe and produces similar acute and chronic hemodynamic benefits in people with HFpEF.

### Vascular stiffening and dysfunction

In addition to impaired contractile reserve, inadequate vasodilation seems to contribute to the inability to reduce end-systolic volume and increase stroke volume in patients with HFpEF (26). Attenuated reductions in mean systemic vascular resistance and effective arterial elastance, increases in pulse wave velocity and arterial elastic moduli, and impairments in aortic distensibility with exercise, which are all associated with the severity of exercise disability, have been observed in patients with HFpEF (27). People with HFpEF frequently display increased arterial stiffness and reduced central aortic compliance (27). This increases the lability of blood pressure swings in HFpEF with changes in fluid volume or vasodilator medicine use (26). Patients with greater arterial stiffening display greater elevation in LV filling pressures and more depressed cardiac output reserve during exercise (26). As such, management of blood pressure can be very challenging in HFpEF, with patients oscillating between severe uncontrolled hypertension and hypotension from day to day.

Patients with HFpEF display endothelial dysfunction compared with age-matched controls. Endothelium-dependent vasodilation is impaired in HFpEF, and the presence and severity of endothelial dysfunction is associated with more severe HF symptoms, worse exercise capacity, and higher event rates (27). Just as systolic dysfunction contributes to impairment in LV diastolic suction, cross-talk occurs between vascular stiffening and diastolic reserve. Acute increases in arterial pressure prolong relaxation, particularly in failing hearts (27).

### Chronotropic reserve and autonomic tone

Chronotropic incompetence, broadly defined as the inability of the heart to increase its rate according to increased activity or demand, is common in patients with cardiovascular disease, produces exercise intolerance, which impairs quality-of-life, and is an independent predictor of major adverse cardiovascular events and overall mortality (28). Chronotropic incompetence is extremely common in HFpEF, with reported prevalence of 57 to 77%. The chronotropic reserve is depressed in HFpEF (27) even compared with older, age-matched controls and independent of rate-slowing medication use. Similar to HFrEF (28), this is likely related to downstream deficits in β-adrenergic stimulation, because the increase in plasma catecholamines with exercise is identical in HFpEF and healthy controls (29). Cardiac output is equal to the product of stroke volume and heart rate, and the inability to augment heart rate with exercise, together with the known impairment in stroke volume reserve in HFpEF, significantly limits cardiac output responses to exercise in many patients (30). While chronotropic incompetence is common in HFpEF, there is no evidence at this time that rate-adaptive pacing is beneficial in patients with HFpEF (8).

Evidence exists for abnormalities in autonomic balance in HFpEF. In an early study, cardio acceleration during the initial phase of exercise, which is driven predominantly by the withdrawal of parasympathetic tone, was blunted in patients with HFpEF (29), although heart rate deficits have been reported only at peak exercise in most subsequent studies (27). Heart rate recovery, defined as the reduction in heart rate after cessation of activity, is also frequently abnormal in patients with HFpEF (31). This marker is related to autonomic tone, as patients with excessive sympathoexcitation and impaired parasympathetic tone have a slower reduction in heart rate after exercise compared with healthy controls. This abnormality in heart rate recovery is independently associated with adverse outcome.

### Peripheral factors

In normal individuals, the degree to which peripheral oxygen extraction (i.e., arterio-mixed venous O_2_ content difference, [C(a-v)O_2_]) increases in response to exercise (≅2.5×) (32) is much greater than changes in systolic volume (≅1.3×) and similar to increases in heart rate (≅2.5×) (32). Several previous studies have found that patients with HFpEF are not able to increase heart rate and systolic volume normally during exercise (33), which implies a greater reliance on the ability to increase C(a-v)O_2_ to augment oxygen uptake (VO_2_) (32). One study demonstrates that peak C(a-v)O_2_ was a significant determinant of exercise capacity in HFpEF (34). The essential functional limitation imposed by impaired O_2_ extraction may reflect intrinsic abnormalities in skeletal muscle or peripheral microvascular function and represents a potential target for therapeutic intervention (34). How to reconcile these conflicting results is not clear at this time; however, they underscore the substantial pathophysiological heterogeneity within the spectrum of HFpEF, and point to the important need for improved methods to individualize therapies to specific phenotypes (30).

Lower extremity skeletal muscle has been found to display increased intramuscular fat content in patients with HFpEF compared with age-matched control individuals, the extent of which was inversely correlated with exercise capacity. Also, morphological and histochemical changes in skeletal muscle have also been described in HFrEF, including marked abnormalities in skeletal muscle mass, composition, capillary density, fiber type, oxidative metabolism, mitochondrial mass, and mitochondrial function (35). Some of these abnormalities in the skeletal muscle have also been observed in cardiac muscle, suggesting the presence of a systemic process (36). Intriguingly, improvements in physical capacity noted with exercise training appear to be mediated not by the heart, but rather by improvement in these abnormalities that are peripheral to the heart in the muscle and vasculature. In addition, another study indicated that the proportion of lean body mass and leg mass in HFpEF was reduced compared to age-matched control individuals (37). Anemia is a common comorbidity in older adults with HFpEF and is associated with worse outcomes (35). Anemia impairs oxygen-carrying capacity, and the severity of anemia predicts mortality, but the role of treatment is uncertain (35). One study demonstrated that administration of epoetin alfa to older adult patients with HFpEF compared with placebo did not change LVEDV, LV mass, nor improved submaximal exercise capacity or quality of life (35).

As described above, peripheral endothelial dysfunction has been reported in HFpEF (27) and might impair dynamic flow-mediated dilatation responses during exercise while also impairing matching of perfusion to regional demand in skeletal muscle microcirculation (35).

## Combined factors for cardiovascular reserve limitation

Obviously, HFpEF is not merely caused by one pathophysiological factor, but in fact is a complex, highly integrated, multisystem loss of cardiac and vascular reserve capacity affecting the left and right ventricles, diastolic and systolic function, atrial reserve, heart rate and rhythm, autonomic control, the vasculature and microcirculation, and the periphery (4). Patients with HFpEF typically display a conglomeration of several reserve impairments that combine to cause symptomatic HF, but the dominant contributors can differ from patient to patient (27). For now, it remains unclear what processes lead to the cardiac, vascular, and peripheral limitations that cause the clinical syndrome of HFpEF (4). However, it is clear from epidemiological studies that the leading risk factors for HFpEF are older age, systemic hypertension, obesity and sedentary lifestyle, and myocardial ischemia, which seem to interact with cardiovascular aging to promote the transition to symptomatic HFpEF (4). Improved understanding of how these risk factors affect the heart and vasculature might improve our understanding of combined reserve limitation in HFpEF (13).

### Cardiac aging

Recent studies have defined aging as an essential factor in the HFpEF epidemic (8). Aging may contribute independently to deterioration of diastolic function (4). Specific alterations in structure and function in aging, such as ventricular arterial stiffening, vascular dysfunction, impaired Ca^2+^ regulation, decreased β-adrenergic reserve, and physical deconditioning, have been identified as critical contributing causes for HFpEF (4). As observed by Borlaug et al. (5), LV stiffness increases with normal aging, despite excellent control of blood pressure and reductions in LV mass. Normal aging is associated with many of the same abnormalities that develop in patients with HFpEF, including diastolic dysfunction, loss of systolic and diastolic reserve, vascular stiffening, and chronotropic incompetence. The cardiac aging process might be accelerated in people with HFpEF (4), and studies suggest that this acceleration is enhanced in women and with weight gain (5). In addition to passive chamber stiffness, diastolic relaxation also becomes compromised with aging, impairing the effects of diastolic suction (5). Aging is also associated with impaired endothelium-dependent vasodilatation. In HFpEF, these combined limitations are exaggerated compared to normal aging (4).

Physiological cardiac aging is associated with an increase in cardiac fibrosis, LV hypertrophy, valvular degeneration, and mainly diastolic dysfunction (38). MicroRNAs (miRNAs) are endogenous small noncoding RNAs, 20–23 nucleotides in length, which have emerged as important post-translational regulators of numerous cardiovascular processes, from myocardial infarction to cardiac aging (39). Numerous miRNAs have been described to be differently expressed and to regulate different cell types and pathways during cardiac aging (38). More recently, studies have revealed that miRNA-34a has been implicated in cardiac aging and might have an important role in cardiac aging via effects on apoptosis, DNA damage, and telomere shortening (38). It was demonstrated that, in HFrEF, microRNA-21 (miR-21) could inhibit the apoptosis of cardiac fibroblasts, leading to cardiac hypertrophy and myocardial fibrosis, but the role of miR-21 in HFpEF remains unknown. A recent study suggested that miR-21 promoted the development of HFpEF by up-regulating the expression of anti-apoptotic gene Bcl-2 and thereby suppressing the apoptosis of cardiac fibrosis (39).

Although our understanding of these processes in the human heart is still in its infancy, cardiac aging may also involve autophagy, a process by which by-products of cell damage are cleared (38). A new study shows that administration of growth/differentiation factor 11 partially reversed age-associated changes in cardiac structure and function in mice.

### Obesity and related comorbidities

Aging seems to be the dominant risk factor for HFpEF. However, obesity and obesity-related comorbidities, such as metabolic syndrome, sedentary lifestyle, and hypertension, are also commonly observed and interact with aging to confer an increased risk of HFpEF (4). Symptoms in HFpEF patients were ascribed to comorbidities that are very frequent among HFpEF patients such as obesity, hypertension, and diabetes. Considering that obesity represents an incubator for other comorbidities (diabetes, hypertension, metabolic syndrome), it is expected that more than 80% of HFpEF patients are overweight or obese (40). Earlier studies suggested that symptoms of dyspnea in obese patients were likely simply related to excess body mass, not cardiac abnormalities (41), but current disease paradigms have begun to embrace the importance of obesity in the pathophysiology of HFpEF, particularly as a cause of systemic inflammation, oxidative stress, and depressed nitric oxide availability that drive cardiac and extracardiac manifestations of disease (41). The increases in blood volume and thus cardiac loading in obesity may cause structural and functional alterations that contribute to HF (40).

Previous studies have reported that subjects with HFpEF may display increased LV diastolic diameter and plasma volume compared to control subjects (41). One study demonstrated that obese subjects with HFpEF had greater estimated plasma volume, LV remodeling, RV enlargement, and increased total heart volume compared to non-obese HFpEF. The LV in obese patients with HFpEF displayed dilation but also an increase in the ratio of LV mass to volume, indicating that concentric remodeling was present (41).

In fact, the role of obesity in HFpEF may be of therapeutic interest (40). Studies have shown that weight gain, increased obesity, and central obesity may accelerate age-related ventricular sclerosis, especially in women (5). Weight loss secondary to bariatric surgery improves diastolic function (40). Besides, long-term exercise-preserving athletes did not exhibit typical age-related loss of LV compliance compared to sedentary individuals, suggesting that fitness can reduce the harmful effects of obesity on the heart, although separating these two components apart is difficult (40). Considering that pharmacological studies were mostly unsatisfactory in patients with HFpEF, a different approach is necessary. In the meantime, weight reduction appears as a good alternative until the medical approach provides a better outcome in this population of the patients (40).

## Diagnosis of HFpEF

HFpEF is a clinical syndrome in which patients have symptoms and signs of HF, a normal or near-normal left ventricular ejection fraction (LVEF ≥50%), and evidence of cardiac dysfunction as a cause of symptoms (e.g., abnormal left ventricular filling and elevated filling pressures) (2). Major HF guidelines reflect reasonable consensus on minimum criteria for the diagnosis of HFpEF while acknowledging diagnostic challenges (2).

**Figure 1:**
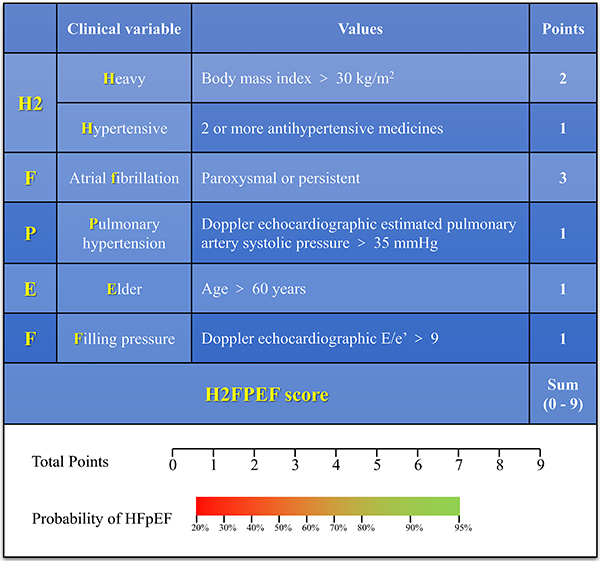
Description of the H2FPEF score and point allocations for each clinical characteristic (top), with associated probability of having heart failure with preserved ejection fraction (HFpEF) based on the total score as estimated from the model (bottom).

The latest report from Reddy et al. (42) reveals a simple and evidence-based way to diagnose HFpEF. There are three main steps in this method: 1) identification of patients with suspected HFpEF based upon clinical evaluation, including history, physical examination, and echocardiography; 2) use of the H2FPEF score to estimate the probability of presence of HFpEF versus non-cardiac causes of symptoms (42); and 3) if H2FPEF score is intermediate (or low but the diagnosis remains uncertain), further testing (including natriuretic peptide level and/or right heart catheterization) is indicated. This approach is displayed in Figure 1.

### Identification of patients with suspected HFpEF

Clinical manifestations of HFpEF are the same as those for general HF, including HFrEF. By far, dyspnea (including dyspnea on exertion, paroxysmal nocturnal dyspnea, or orthopnea) and fatigue are the most common symptoms. Physical signs of HF (such as elevated jugular venous pressure, pulmonary rales, and lower extremity edema) may or may not be present (43).

Echocardiography is a key component of the diagnosis and evaluation of patients with suspected HF. Evaluation of patients with HF includes Doppler transthoracic echocardiography to evaluate LVEF, estimate pulmonary artery systolic pressure, assess left ventricular filling pressure, and assess cause of HF (43). However, if the LVEF cannot be adequately assessed by echocardiography, alternative methods including cardiovascular magnetic resonance, cardiac radionuclide ventriculography, and cardiac computed tomography are suggested (42).

Causes of the clinical syndrome of HF with an LVEF ≥50% other than HFpEF include a cardiomyopathy (e.g., hypertrophic or restrictive cardiomyopathy), cardiac amyloidosis, significant valve disease (severe stenosis or regurgitation or at least moderate mixed stenosis and regurgitation), pericardial disease (e.g., constrictive pericarditis), and high-output HF (42). Clinical evaluation including echocardiography is helpful in identifying these conditions (42).

From the above, HFpEF should be suspected in individuals with all three of the following features: 1) one or more symptoms of HF such as dyspnea or fatigue; physical signs of HF may or may not be present; 2) an LVEF ≥50%; and 3) no apparent cause of HF symptoms other than HFpEF.

### Estimation of HFpEF probability using the H2FPEF score

**Figure 2:**
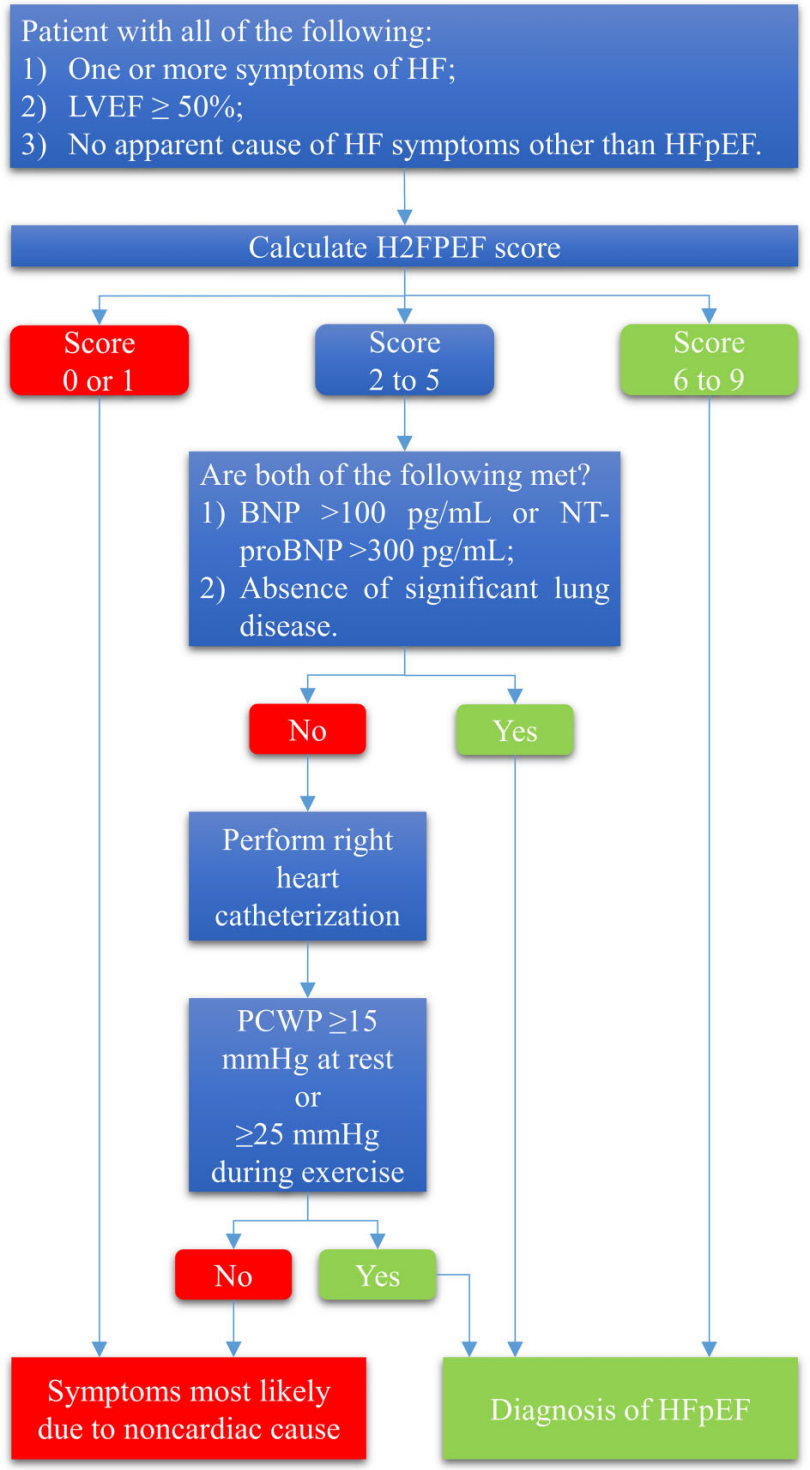
Approach to diagnosis of heart failure with preserved ejection fraction (HFpEF). LVEF: left ventricular ejection fraction; BNP: brain natriuretic peptide; NT-proBNP: N-terminal pro-brain natriuretic peptide; PCWP: pulmonary capillary wedge pressure.

In patients with suspected HFpEF, we suggest using the H2FPEF score to estimate the probability of HFpEF versus non-cardiac causes of dyspnea. This clinically validated score is the sum of the points based on the following clinical variables (42): 1) Heavy: body mass index >30 kg/m^2^ (two points); 2) Hypertensive: two or more antihypertensive medicines (one point); 3) Atrial fibrillation (AF): paroxysmal or persistent (three points); 4) Pulmonary hypertension (PH): pulmonary artery systolic pressure >35 mmHg using Doppler echocardiography (one point); 5) Elder: age >60 years (one point); 6) Filling pressure: Doppler echocardiographic E/e' >9 (one point) (Figure 2).

The probability that HFpEF is the cause of symptoms increases with increasing total H2FPEF score (range 0 to 9). A low H2FPEF score of 0 or 1 is associated with a low (<25%) probability of HFpEF. An intermediate H2FPEF score of 2 to 5 is associated with an intermediate (40 to 80%) probability of HFpEF. A H2FPEF score of 6 or greater is associated with a greater than 90% probability of HFpEF and is thus considered diagnostic for HFpEF.

### Further testing for low or intermediate H2FPEF score patients

A low score of 0 or 1 suggests that symptoms are most likely due to a non-cardiac cause, and such reasons should be investigated (42). However, if the cause of symptoms remains uncertain after evaluation for non-cardiac causes, cardiology consultation and right heart catheterization are suggested to determine if HFpEF is present (42).

With an intermediate H2FPEF score of 2 to 5, we should take further steps to assess the following: 1) Is the natriuretic peptide level high (BNP >100 pg/mL or NT-proBNP >300 pg/mL)?; 2) Is there an absence of significant lung disease? If the above criteria are satisfied, then HFpEF can be diagnosed. If any of the above or both are not met, we suggest cardiology consultation and right heart catheterization (42).

Right heart catheterization is not universally required for diagnosis and evaluation of HFpEF (42). However, in selected patients with intermediate H2FPEF scores (and selected patients with low H2FPEF score with undetermined causes of symptoms), cardiology consultation and right heart catheterization for assessment of cardiac filling pressures at rest and exercise is the clinical gold standard to make or exclude the diagnosis of HFpEF (42). On right heart catheterization, pulmonary wedge pressure (PCWP) ≥15 mmHg at rest or ≥25 mmHg during exercise is diagnostic for HFpEF. Pressures are measured at end-expiration. Exercise is performed during right heart catheterization with cycle ergometry (in patients with internal jugular venous access) or arm abduction with weights (in those with femoral venous access) (14).

### Evidence and limitations of the H2FPEF score method

The H2FPEF score was derived from data of 414 patients with an LVEF ≥50% (267 with HFpEF confirmed by pulmonary capillary wedge pressure and 147 with non-cardiac dyspnea) and validated in a test cohort of 100 patients (61 with HFpEF) (42). The odds of HFpEF doubled for each 1-unit H2FPEF score increase (odds ratio 1.98; 95%CI: 1.74–2.30), with an area under the curve of 0.841. The H2FPEF score was superior to an algorithm based on expert consensus (increase in area under the curve of 0.169; 95%CI: 0.120–0.217). In the independent test cohort, the area under the curve was 0.886. We suggest the use of the H2PEF score as a general guide in patients with suspected HFpEF while recognizing the limitations of this score (42). The score has not been validated in more extensive and diverse populations. The score includes two elements from echocardiography (estimated pulmonary artery systolic pressure [PASP] and E/e' ratio), which are subject to inaccurate results with suboptimal image acquisition and interpretation (42).

## Treatment and prognosis of HfpEF

Clinical trials in HFpEF have produced neutral results to date and treatment is largely directed toward associated conditions (e.g., hypertension) and symptoms (e.g., edema). This approach is consistent with recommendations for treatment of patients with HFpEF included in the 2013 American College of Cardiology Foundation/American Heart Association (ACC/AHA) HF guidelines (44). The following two strong recommendations were included: 1) Systolic and diastolic hypertension should be controlled in accordance with published clinical practice guidelines to prevent morbidity; 2) Diuretics should be used to relieve symptoms due to volume overload. Similar recommendations were included in the 2016 European Society of Cardiology HF guidelines (2).

The management of HFpEF differs from the management of HFrEF (45). The results of clinical trials have demonstrated that while neurohumoral antagonists such as beta-blockers, angiotensin-converting enzyme (ACE) inhibitors, and angiotensin receptor blockers (ARBs) as well as cardiac resynchronization are effective in HFrEF, these therapies do not decrease morbidity and mortality in HFpEF (45). These data suggest that there are fundamental differences in the pathophysiology underlying HFrEF and HFpEF. All the therapies that improve mortality in HFrEF also reverse the LV dilatation in HFrEF. Since patients with HFpEF have no or minimal LV dilatation, these agents are not as effective in HFpEF. Although asymptomatic diastolic dysfunction is a risk factor for the development of HFpEF and mortality, data are lacking on the efficacy of therapy to reduce the risk of progression to HFpEF(45).

Diastolic function worsens as part of aging even in individuals without other forms of cardiovascular disease (5). Asymptomatic diastolic dysfunction is a predictor of future cardiovascular morbidity, but prognosis differs from that in patients with symptoms of HFpEF (5).

In this section, we will discuss the HFpEF treatment around the management of associated conditions and pharmacologic therapy. As for the prognosis of HFpEF, we will discuss it in detail using the latest clinical trials.

### Management of associated conditions

A key component in the treatment of HFpEF patients is treating the contributing factors and the comorbidities of the disease (46). This method plays a significant role in the clinical course of the disease (46). One study has pointed out that the most common comorbidities include hypertension, lung disease, coronary artery disease, atrial fibrillation (AF), obesity, anemia, diabetes mellitus, kidney disease, and sleep-disordered breathing. These comorbidities have a significant implication for the clinical course, and the vast majority of subsequent hospitalizations in patients with HFpEF are not because of HF (46).

Hypertension remains one of the major modifiable risk factors in HFpEF development and progression. Of nearly 400 cases of new HF in the Framingham study, 91% were preceded by the development of hypertension (46). Treatment of hypertension has been shown to prevent the development of HF in several clinical trials, particularly among the elderly (47). Reduced incidence of HF in post-menopausal women has also been associated with markers of healthy lifestyles including high-quality diet, increased physical activity, maintenance of healthy body weight, and lack of tobacco use, which are similar to the non-pharmacological treatment recommendations for hypertension. The choice of a specific antihypertensive agent must be individualized in the presence of coexisting diseases such as diabetes mellitus or chronic obstructive pulmonary disease. However, there may be class-specific effects. In an ancillary analysis of data from the ALLHAT trial, chlorthalidone reduced the incidence of new-onset HFpEF compared with amlodipine, lisinopril, and doxazosin, whereas both lisinopril and chlorthalidone were effective in reducing the incidence of HFrEF (47). Diuretics or venodilators, such as nitrates, should be used with caution. Patients with a small, stiff left ventricular chamber are particularly susceptible to excessive preload reduction, which can lead to underfilling of the left ventricle, a fall in cardiac output, and hypotension (46). LV hypertension is frequently present in patients with diastolic dysfunction. Regression of LV hypertension is an important therapeutic goal since diastolic function may be improved. Studies utilizing a variety of agents such as beta-blockers, diuretics, and calcium channel blockers demonstrated regression of LV hypertension, though medications targeting the renin-angiotensin-aldosterone system (RAAS) led to higher rates of LV hypertension reversal. The optimal therapy of hypertension in patients with HFpEF (i.e., diastolic dysfunction) is uncertain. The management of hypertension is a cornerstone of HFpEF management, and careful matching of antihypertensive treatments to patient phenotype holds great promise for improving outcomes in patients with HFpEF (46).

Atrial fibrillation (AF) and HF often co-exist. The presence of one increases the likelihood of the other and each can cause the other (48). AF is common in HFpEF, identified at some point in two-thirds of patients, and its presence is associated with increased morbidity and mortality. AF can impair myocardial function by multiple mechanisms and HF may result or worsen. Most observational studies evaluating the impact of AF in patients with HF, and the converse, were performed many years ago (46). They present conflicting data as to whether AF is an independent predictor of mortality in patients with HF. As suggested in the 2013 ACC/AHA HF guidelines, AF is managed in patients with HFpEF according to published clinical practice guidelines to improve symptomatic HF (44). For the AF treatment in HFpEF patients, rhythm control is prior to rate control. Rhythm can be controlled with antiarrhythmic drug therapy, catheter ablation, or surgical ablation, which is the preferred approach in patients with HF who are hemodynamically unstable or who are persistently symptomatic despite adequate rate control. Surgical ablation is the treatment of choice in patients with recent-onset AF in whom there is an exacerbation of HF even if rate control is achieved (48). Most often, the efficacy of successful restoration and long-term maintenance of sinus rhythm is dependent in part on how long a patient has been in persistent AF, but several other predictors exist including left atrial size. Both antiarrhythmic drug therapy and catheter ablation are available to achieve this end in select patients (48). Rate control to prevent rapid AF acutely and/or chronically usually leads to an improvement in symptoms in patients with HF. In addition, slowing of the ventricular rate often leads to a moderate or, in some cases, marked improvement in left ventricular function. Beta-blockers and calcium channel blockers are the usual first-line agents. For patients who cannot receive a beta-blocker due to issues such as bronchospasm, a non-dihydropyridine calcium channel blocker may be used. Digoxin should be used more cautiously (48). An important component of the management of AF, regardless of whether rhythm control or rate control is chosen, is anticoagulation drug use to prevent systemic embolization.

Myocardial ischemia in HFpEF can result from epicardial coronary artery disease (CAD), high wall stress, or microvascular dysfunction (36). CAD is common among patients with HFpEF. As an example, a series of patients with HFpEF reported that two-thirds of patients had anatomically significant CAD (45). The presence of CAD was an independent predictor of increased mortality, along with more considerable deterioration in LV systolic function over time. Patients with HFpEF and symptoms and signs of ischemia are treated with standard therapy. Beta-blockers are preferred for initial treatment and prevention of anginal symptoms (45). Calcium channel blockers and long-acting nitrates are alternatives if beta-blockers are contraindicated or cause side effects; they can also be added as combination therapy if monotherapy is not successful (45). Short-acting nitrates are used for immediate angina relief. Patients with coronary artery disease with drug-resistant ischemic HFpEF may require coronary revascularization by percutaneous coronary intervention or coronary artery bypass graft surgery (44). In a single-center, retrospective series, revascularization was associated with improved survival and less deterioration in EF (45). However, prospective trial data are not available regarding the effects of revascularization in HFpEF. The optimal management also requires periodic evaluation (every 6 to 12 months) of the patient's clinical status, using the history, physical examination, and on occasion, the electrocardiogram (45).

Hyperlipidemia is the abnormally elevated levels of any or all lipids or lipoproteins in the blood. Treatment of lipid levels is recommended for the primary and secondary prevention of cardiovascular disease (45). Two large randomized trials found that statins do not have a beneficial effect in patients with HFrEF (49). However, observational data suggest that statins might be of benefit in patients with HFpEF (49). Randomized trials are required to confirm these observations (45). We recommend the use of statins in patients with HFpEF who have an indication for statin therapy.

### Pharmacologic therapy

Treatment of HFpEF is mostly governed by the management of associated conditions and symptoms since there is limited direct evidence to support a specific drug regimen. Based on the available evidence, we suggest treatment with a mineralocorticoid receptor antagonist in patients with HFpEF who can be appropriately monitored. Diuretics are used to treat volume overload, but as noted above, care must be taken to avoid volume depletion (50). Other medications such as ARBs, ACE inhibitors, calcium channel blockers, and beta-blockers are used as treatment for hypertension, but lack proven efficacy to alter clinical outcomes in HFpEF (51). We recommend against the use of phosphodiesterase-5-inhibitors, organic nitrates such as isosorbide, or digoxin (aside from use for ventricular rate control in atrial fibrillation) to treat HFpEF (52).

For patients with clear evidence of HFpEF (including increased brain natriuretic peptide [BNP] or NT-proBNP) who can be carefully monitored for changes in serum potassium and renal function, we suggest treatment with a mineralocorticoid receptor antagonist. The serum potassium should be <5.0 mEq/L and estimated glomerular filtration rate should be ≥30 mL·min^−1^·(1.73 m^2^)^−1^. Evidence to support this approach comes from the Treatment of Preserved Cardiac Function HF with an Aldosterone Antagonist (TOPCAT) trial (52,53).

Diuretics improve symptoms of HF patients and are widely used irrespective of LVEF (52). Loop diuretics are the primary treatment for reducing congestive symptoms associated with hypervolemia. However, in HFpEF, maintaining optimal volume status is often difficult. Patients with HFpEF are highly sensitive to volume changes and generally have a narrow window between volume overload, causing congestive symptoms, and hypovolemia. Overly aggressive diuresis may result in further reductions in cardiac output, hypotension, and decreased renal function (52). The beneficial effect of diuretics was suggested by an ancillary study from the CHAMPION trial, in which medical treatment decisions driven by the knowledge of pulmonary artery pressure data were associated with a significant reduction in hospitalizations for HF (50). The majority of medication changes were in diuretic usage, and mean diuretic dose increased significantly more in the pulmonary artery pressure-guided treatment group. These data provide indirect evidence supporting the efficacy of diuretics to reduce morbidity in HFpEF.

Evidence of efficacy of beta blocker therapy in patients with HFpEF is lacking. An individual patient-level meta-analysis of 11 randomized controlled trials of beta blockers in patients with HF found no evidence of benefit in the small subgroup of patients in sinus rhythm with LVEF ≥50% (51). There was no consistent benefit from beta blockers among patients with atrial fibrillation. The effects of beta blockers in patients in sinus rhythm varied according to baseline LVEF: for patients with baseline LVEF <40%, beta blocker therapy significantly reduced all-cause mortality; for patients with baseline LVEF of 40 to 49%, all-cause mortality was nominally but not statistically significantly lower with beta blocker therapy; for patients with baseline LVEF of ≥50%, beta blocker therapy did not reduce all-cause mortality (51). We suggest not using beta blockers for HFpEF in the absence of an alternative indication, such as angina.

Hypertension is one of the leading causes of HFpEF in older adults and calcium channel blockers (CCB) is one of the commonly prescribed anti-hypertensive drugs (52). Because there is currently no evidence-based guideline recommendation for the use of CCBs in HFpEF, these drugs were likely used for the control of blood pressure and heart rate. These findings suggest that the negative inotropic and chronotropic effects of CCBs had no negative association with outcomes in HFpEF (52). CCBs have been shown to have variable effects on cardiovascular outcomes in HF patients. One study demonstrated that, in real-world hospitalized older HFpEF patients not receiving prior CCBs, a new discharge prescription for CCBs had no associations with the primary composite endpoint of total mortality or HF hospitalization and individual endpoints of mortality or hospitalization, regardless of the class of CCBs (54). CCBs may also be useful in the treatment of hypertension in patients with HFpEF, though the evidence is very limited (55). CCBs are generally used as a third- or fourth-line antihypertensive in HFpEF patients with severe hypertension (54). In addition, as discussed separately, in patients with hypertrophic cardiomyopathy, verapamil may improve symptoms and measures of LV diastolic function.

There is no evidence from randomized clinical studies that ACE inhibitor therapy directly improves overall morbidity or mortality in patients with HFpEF (56). Because patients with HFpEF frequently have comorbidities such as renal insufficiency, ACE inhibitors should be used carefully to avoid the risk of renal dysfunction and hypotension (56). Despite these concerns, ACE inhibitors play an important role in the treatment of the disease processes that contribute to the development of HFpEF, namely hypertension, coronary heart disease, diabetes, and chronic kidney disease (56). ACE inhibitors are beneficial in hypertensive heart disease. The reduction in systemic pressure can theoretically lead to regression of LV hypertension and a gradual improvement in diastolic function. The clinical efficacy of an ACE inhibitor in patients with HFpEF was assessed in the PEP-CHF trial in which 850 patients ≥70 years of age had diastolic dysfunction: 79% had a history of hypertension; patients with substantial LV systolic dysfunction or valve disease were excluded. Overall, there was no impact of ACE inhibitor on the primary endpoint (56). A *post hoc* analysis at one year found that treatment with perindopril was associated with an almost significant trend toward reduction in the primary endpoint of combined all-cause mortality and unexpected hospitalization for HF; this effect was entirely due to fewer unexpected hospitalizations for HF. The patients treated with perindopril also had significant improvements in functional class and six-minute walk distance (56).

ARBs, like ACE inhibitors, help blunt the adverse cardiovascular effects of angiotensin II. However, ARBs exert their effect further downstream and block the association of angiotensin II with its receptor AT1 (57). There is no evidence from randomized clinical studies that ARB therapy directly improves overall morbidity or mortality in patients with HFpEF. There is no evidence of improved diastolic function with ARB treatment compared with other therapies in patients with asymptomatic LV diastolic dysfunction or overt HFpEF (57). Two large, randomized, double-blind, placebo-controlled trials have evaluated morbidity and mortality outcomes with ARB use in the HFpEF population. The first one is CHARM-Preserved trial, which demonstrated a moderate benefit for HF hospitalizations in the use of ARBs in the HFpEF population (57,58). The I-PRESERVE trial, that followed the CHARM-Preserved trial, failed to support the potential improvement in clinical outcomes that CHARM-Preserved demonstrated (59). Besides, two other small trials have examined ARB use in patients with HFpEF. Both studies examined the effects of an ARB (losartan or valsartan) on exercise tolerance in patients with HFpEF and demonstrated conflicting results compared to placebo (60).

We recommend against the use of organic nitrates to treat HFpEF. Evidence of efficacy is lacking and a randomized trial found that use of isosorbide mononitrate tended to reduce activity levels in patients with HFpEF (61). There are many clinical trials that have proven that phosphodiesterase-5 inhibitors have no benefit for HFpEF. Based on the results of these trials, we do not use phosphodiesterase-5 inhibitors for the treatment of HFpEF (23). The DIG ancillary trial, a parallel study to the DIG trial, evaluated the role of digoxin in patients with HF and an LVEF >45% (62). At a mean follow-up of 37 months, digoxin had no effect on all-cause or cause-specific mortality, or all-cause or cardiovascular hospitalization (62). We recommend against the use of digoxin to treat patients with HFpEF except for atrial fibrillation with poorly controlled ventricular rate.

### Prognosis

The prognosis of patients with HFpEF is less well defined than that of patients with HFrEF. Population-based data from hospitalized patients have shown similar outcomes in patients with HFpEF and HFrEF (63). However, a large meta-analysis, including community-based studies and trials, observed lower mortality in HFpEF compared to HFrEF, though survival was still much worse than in people without HF (63,64). Since diastolic dysfunction is common in subjects in the age group at risk for HFpEF, it is possible that HFpEF may be over-diagnosed in patients with echocardiographic evidence of diastolic dysfunction and a clinical syndrome that mimics HF (but not due to HF) such as pulmonary disease, obesity, kidney disease, or deconditioning (63).

Among patients hospitalized for HF, the mortality rates are high but the data are again conflicting as to whether or not the prognosis is different in HFpEF and HFrEF (63). Among 6076 patients discharged from a Mayo Clinic Hospital in Olmsted County, Minnesota with a diagnosis of decompensated HF over a 15-year period (1987 to 2001), 53% had HFrEF and 47% had HFpEF. One-year mortality was relatively high in both groups but slightly lower in patients with HFpEF (29 versus 32% in patients with HFrEF, adjusted HR 0.96, 95%CI: 0.92–1.00). Survival improved over time for those with HFrEF but not for those with HFpEF. In a prospective evaluation of 413 patients hospitalized for HF, the relative risk for six-month mortality was lower for HFpEF than for HFrEF (13 *vs* 21%, adjusted HR 0.51). In a cohort of 2802 patients discharged from 103 hospitals in Ontario with a diagnosis of decompensated HF, one-year mortality was 22% in patients with HFpEF *vs* 26% in patients with HFrEF. This difference was not statistically significant (63).

Independent predictors of mortality in patients with HFpEF in different studies include older age, male gender, New York Heart Association (NYHA) class, lower LVEF, the extent of coronary artery disease, peripheral artery disease, diabetes, impaired renal function, the degree of diastolic dysfunction as assessed by Doppler echocardiography, elevated plasma natriuretic peptide levels, pulmonary hypertension, RV dysfunction, and increased red cell distribution width (63).

The proportions of cardiovascular and non-cardiovascular deaths among patients with HFpEF have varied among trials and epidemiologic studies, with higher proportions of non-cardiovascular deaths in population-based studies (65). The mode of death was evaluated in patients with symptomatic HFpEF (NYHA class II to IV HF with LVEF ≥45%) enrolled in the I-Preserve trial (66). The annual mortality rate was 5%. Sixty percent of deaths were cardiovascular (26% sudden death, 14% HF, 5% myocardial infarction, and 9% stroke), 30% were non-cardiovascular (including cancer and infection/sepsis), and 10% were of unknown cause. Irbesartan treatment did not affect the mortality rate or the distribution of mode of death. In a community-based study that did not include trial participants, the rate of non-cardiovascular death was substantially higher, likely reflecting the greater frailty and higher comorbidity burden seen in patients in the general population compared with trial participants (63,67)

Morbidity outcomes in HFrEF and HFpEF are similar. These include the rate and frequency of hospitalization for HF, symptomatic status as measured by abnormalities in myocardial oxygen consumption, six-minute walk distance, Minnesota Living with HF Questionnaire scores, and other quality-of-life indicators. Therefore, patients with HFpEF have a morbidity burden equivalent to that of patients with HFrEF (63).

## Summary

**Figure 3:**
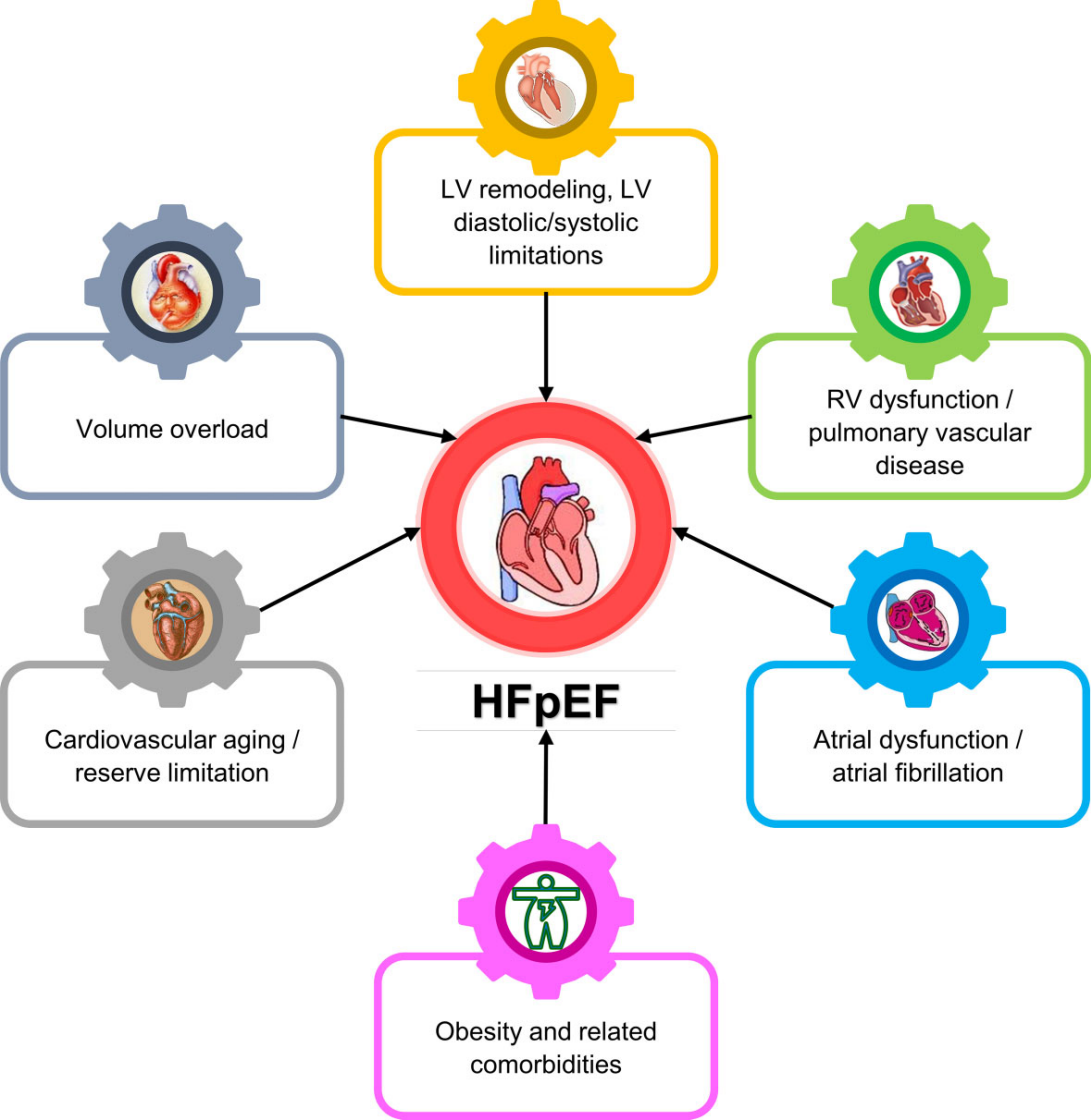
Mechanisms in heart failure with preserved ejection fraction (HFpEF) outlined in this review. LV: left ventricle; RV: right ventricle.

In particular, we should note that HFpEF and diastolic dysfunction are not synonymous. LVEF is an essential clinical indicator. Patients with HFpEF have LVEF ≥50%, usually with standard heart size and typically exhibit concentric remodeling or hypertrophy. The most apparent and common abnormality in HFpEF patients is associated with diastolic dysfunction. Diastolic dysfunction may coexist with slack damage or increased chamber stiffness or both. These abnormalities, in turn, lead to difficult breathing. In addition to diastolic dysfunction, patients with HFpEF also exhibit limitations such as systolic dysfunction (68), contractile reserve (69), pulmonary hypertension (70), right ventricular dysfunction (71), vascular and endothelial abnormalities (72), left atrial dysfunction (20), and peripheral abnormalities (73). Patients with HFpEF often have associated comorbidities such as hypertension and metabolic syndrome contributing to impaired endothelial function. This, in turn, adversely remodels aortic and downstream arterial hemodynamics, worsening the pathophysiology in HFpEF (73). The complex interaction of all these pathophysiological mechanisms is responsible for exacerbating symptoms and worsening HFpEF results. Changes in ventricular, vascular, and peripheral structures and functions leading to HFpEF are thought to be associated with aging and co-morbidities common in HFpEF, including hypertension, obesity, insulin resistance, sedentary lifestyle, and coronary artery disease. This interaction may be mediated by low levels of inflammation and loss of availability of nitric oxide (Figure 3).

About half of all HF patients worldwide have LVEF ≥50%. The main contributors to HFpEF are systemic hypertension, aging, coronary artery disease, diabetes, metabolic syndrome, obesity, and kidney disease. Occult coronary heart disease is a common and potentially reversible cause of HFpEF. In the diagnosis of HFpEF, it is vital to exclude mimics including non-HF conditions and other causes of HF with LVEF ≥50%, such as valvular heart disease, pericardial disease, cardiomyopathy, cardiac amyloidosis, and high output HF. Regarding the diagnostic approach, we highly recommend H2FPEF score diagnostic method from Barry A Borlaug's latest study (42), which is simple, efficient, and easy to implement clinically. In his procedure, natriuretic peptide levels are helpful in the differential diagnosis of patients with a moderate probability of HFpEF.

An essential component in the treatment of HFpE is the treatment of contributing factors and comorbidities that often occur and significantly affect clinical processes. The most common include high blood pressure, lung disease, coronary artery disease, obesity, anemia, diabetes, kidney disease, and sleep-disordered breathing. The general principles for the treatment of HFpEF are to control pulmonary congestion and peripheral edema, treat systolic hypertension, prevent rapid heart rate (especially in patients with atrial fibrillation), and establish coronary revascularization in patients with coronary heart disease. Diuretics or intravenous dilators must be used with caution in patients with left ventricular diastolic dysfunction with a small, stiff left ventricle. When atrial fibrillation occurs in patients with HFpEF, restoration and maintenance of sinus rhythm is preferred, followed by rate control. We do not recommend the use of beta-blockers to treat HFpEF without alternative indications such as angina. For patients with clear HFpEF evidence (including increased BNP), we recommend monitoring changes in serum potassium and renal function and treatment with mineralocorticoid antagonists. We do not recommend the use of organic nitrates, phosphodiesterase-5 inhibitors, or digoxin (except for ventricular rate control of atrial fibrillation) for the treatment of patients with HFpEF. Exercise training is the only intervention that can continuously improve HFpEF's functional capacity and quality of life. The morbidities of HFpEF patients are almost identical to that of HFrEF patients. Mortality rates for both HFpEF and HFrEF are high; so far, published data on mortality differences are contradictory.

HFpEF is a complex disorder caused by multifactorial stresses secondary to comorbidities. The current challenge is finding new multidirectional strategies to abrogate cardiac remodeling. Exploring detail pathophysiological mechanisms, seeking easy and popular diagnostic approaches, and finding a precise treatment are the main objectives to overcome HFpEF.
