# Erratum notice for: “Heart failure with preserved ejection fraction: an update on pathophysiology, diagnosis, treatment, and prognosis” [Braz J Med Biol Res (2020) 53(7): e9646 | doi: 10.1590/1414-431X20209646]

**DOI:** 10.1590/1414-431X20219646erratum

**Published:** 2021-02-26

The Brazilian Journal of Medical and Biological Research has been informed by the authors that the second affiliation “German Center for Cardiovascular Research (DZHK), Partner Site Berlin, Berlin, Germany” was cited erroneously and should be withdrawn from the published article “Heart failure with preserved ejection fraction: an update on pathophysiology, diagnosis, treatment, and prognosis”.

The correct list of authors and institutions is as follows:

Chao Ma https://orcid.org/0000-0003-1444-4668 ^1^, Huan Luo https://orcid.org/0000-0001-7886-8967 ^2^, Lei Fan https://orcid.org/0000-0003-2084-7869 ^3^, Xiaoyan Liu https://orcid.org/0000-0003-3976-684X ^4^, and Chengshan Gao https://orcid.org/0000-0001-8988-3302 ^4^

^1^Berlin Institute of Health Center for Regenerative Therapies & Berlin – Brandenburg Center for Regenerative Therapies (BCRT), Charité - Universitätsmedizin Berlin, Campus Virchow Klinikum (CVK), Berlin, Germany

^2^Klinik für Augenheilkunde, Charité-Universitätsmedizin Berlin, Corporate Member of Freie Universität Berlin, Humboldt-Universität zu Berlin, and Berlin Institute of Health, Berlin, Germany

^3^Department of Orthopedic Surgery, Henan Provincial People's Hospital, Zhengzhou, Henan, China

^4^Department of Cardiovascular Surgery, Second Affiliated Hospital of Zhengzhou University, Zhengzhou, Henan, China

Correspondence: Chengshan Gao: <chengshangao@yahoo.com>

